# Molecular and Clinical Insights into the Invasive Capacity of Glioblastoma Cells

**DOI:** 10.1155/2019/1740763

**Published:** 2019-07-29

**Authors:** Carlos Velásquez, Sheila Mansouri, Carla Mora, Farshad Nassiri, Suganth Suppiah, Juan Martino, Gelareh Zadeh, José L. Fernández-Luna

**Affiliations:** ^1^Department of Neurological Surgery and Spine Unit, Hospital Universitario Marqués de Valdecilla and Instituto de Investigación Marqués de Valdecilla (IDIVAL), Santander, Spain; ^2^Division of Neurosurgery, Toronto Western Hospital, University Health Network, University of Toronto, Toronto, Canada; ^3^MacFeeters-Hamilton Center for Neuro-Oncology Research, Princess Margaret Cancer Centre, Toronto, Canada; ^4^Genetics Unit, Hospital Universitario Marqués de Valdecilla and Instituto de Investigación Marqués de Valdecilla (IDIVAL), Santander, Spain

## Abstract

The invasive capacity of GBM is one of the key tumoral features associated with treatment resistance, recurrence, and poor overall survival. The molecular machinery underlying GBM invasiveness comprises an intricate network of signaling pathways and interactions with the extracellular matrix and host cells. Among them, PI3k/Akt, Wnt, Hedgehog, and NFkB play a crucial role in the cellular processes related to invasion. A better understanding of these pathways could potentially help in developing new therapeutic approaches with better outcomes. Nevertheless, despite significant advances made over the last decade on these molecular and cellular mechanisms, they have not been translated into the clinical practice. Moreover, targeting the infiltrative tumor and its significance regarding outcome is still a major clinical challenge. For instance, the pre- and intraoperative methods used to identify the infiltrative tumor are limited when trying to accurately define the tumor boundaries and the burden of tumor cells in the infiltrated parenchyma. Besides, the impact of treating the infiltrative tumor remains unclear. Here we aim to highlight the molecular and clinical hallmarks of invasion in GBM.

## 1. Introduction

In adults, glioblastoma (GBM) is the most common primary tumor in the central nervous system, with an incidence of 4.5 cases per 100,000 inhabitants. The median survival remains 14 months despite highly aggressive standard treatment protocols [1]. One of the key hallmarks of GBM hindering effective therapy is the diffuse invasiveness of the tumor cells through the normal parenchyma, causing tumor recurrence in close proximity or distant from the original tumor site. This feature appears to be independent of tumor grade, as both higher and lower grade gliomas tend to recur as a result of invasion of tumor cells into surrounding brain tissue [2]. The mechanism of glioma cell invasion involves both biochemical and biophysical processes that regulate cell shape and its movement across the intercellular space, concurrent with rearrangement of the extracellular matrix (ECM). In the recent years several molecular pathways have been associated with glioma invasion and represent potential therapeutic targets and biomarkers for prognosis. Taking this into account, it is mandatory for oncologists, neurosurgeons, neurologists and neuroscientists to be familiar with the most important signaling processes underlying glioma invasion and understand the clinical manifestations of GBM invasion for appropriate treatment planning. Herein, we review key cellular pathways and processes that regulate glioma cell invasion and describe their relevance as potential therapeutic targets for management of gliomas.

## 2. The Molecular Hallmarks of Invasion in GBM

### 2.1. Adhesion Molecules

The first stage of glioma cell invasion is detachment from the surrounding tumor tissue, a process that involves cell surface adhesion molecules such as neuronal cell adhesion molecule (NCAM) and cadherins as key players in this process. It had been demonstrated that cadherin instability leads to glioma cell migration [3] and NCAMs modify the ECM by downregulating the expression of matrix metalloproteinases that degrade cadherins and, thereby, hinder tumor cell motility [4]. Furthermore, the expression of NCAMs is inversely related to glioma grade, which is in agreement with data showing that loss of this molecule enhances tumor cell migration [5]. Recent transcriptomic and proteomic analyses have reproduced these findings and have identified a new splice variant of NCAM1 with potential implications in cell signaling [6].

In addition to NCAMs, intercellular adhesion molecule-1 (ICAM1), a member of the immunoglobulin family of genes and expressed in several cell types, has recently been shown to contribute to glioma cell invasion [7]. ICAM1 is involved in several processes, including inflammatory cell movement, effector leukocyte activity, antigen-presenting cells adhesion to T lymphocytes, and signal transduction pathways through outside-in signaling processes. Upon induction of inflammation, leukocytes interact with ICAM1 on the endothelial cells, which allows them to cross the barrier vessel wall [8]. It has been shown that thalidomide can suppress ICAM1 expression and inhibit invasion mediated by ICAM1 in lung cancer [9]. In glioma, it was shown that radiation increased ICAM1 expression, thereby, promoting migration and invasion of the tumor cells [10]. Lin et al. reported that ICAM1 enhances the invasiveness of GBM cells into the healthy brain tissue and may, therefore, serve as a marker of invasion in GBM [11].

Integrins (ITGs) are another key component of the interface between tumor cells and other cells in the microenvironment and function as receptors that regulate cell adhesion to ECM proteins or cell surface proteins on other stromal cells [12]. They also play a central role in linking extracellular contacts with the intracellular cytoskeleton through two different signaling mechanisms; ITGs cluster in the membrane upon extracellular ligands binding and transduce intracellular signals through their cytoplasmic domain (*β* subunit) by activation of kinases such as Focal Adhesion Kinase (FAK), Integrin-Linked Kinase (ILK) and Rho-GTPases. Through this mechanism, ITGs then activate pathways leading expression of genes that modulate cell proliferation, survival, differentiation, and migration (outside-in signaling)[12]. It is also possible for cytoplasmic proteins to modulate the extracellular affinity of ITGs for their ligands (inside-out signaling) and contribute to cell migration and invasion [13].

ITGs are expressed by various cell types in the tumor microenvironment including endothelial cells, immune cells, and pericytes and promote tumorigenesis. In particular, ITGs regulate invasion and metastasis by providing the traction necessary for cell migration [14]. They also modulate the expression of proteases that play a role in remodelling the ECM. Involvement of several ITGs in epithelial to mesenchymal transition (EMT) has been described. For example, *α*v*β*1 ITG was shown to mediate an EMT-like program in GBM cells [15]. In addition, *α*v*β*3/*α*v*β*5 was shown to promote GBM cell migration and invasion by enhancing the adhesion of tumor cells to components of the ECM via fibronectin, vitronectin, osteopontin, or periostin [16–18] and activation of intracellular signaling pathways such as FAK, Rho-GTPases, Shc/MAP-Kinases, and Src Family Kinases [14, 19, 20]. *α*v*β*3 also enhances GBM invasion through the activation of matrix metalloproteinase 2 (MMP2) at the plasma membrane, which is thought to degrade components of the ECM and enhance cell motility [14]. Finally, inhibition of *α*v*β*3/*α*v*β*5 in mouse models reduces GBM cell migration and invasion [21]. Another study by Delamarre et al. has also shown that *α*6*β*1 is associated with invasive phenotype in U87 GBM cell line* in vitro* and* in vivo* [22]. Therefore, targeting specific ITGs in GBM could inhibit tumor invasion and aggressive features.

### 2.2. ECM Composition and Invasion

ECM composition plays a critical role in the invasion process and the tumor-associated ECM is intrinsically different from the ECM within the normal parenchyma [23, 24]. For instance, hyaluronic acid (HA) enrichment in tumor microenvironment promotes cell invasion through positive feedback regulation of NF*κ*B that may result from ionizing radiation or hypoxia [25–27]. On the other hand, it has been shown that a glycosylated chondroitin sulfate proteoglycans- (CSPGs-) enriched ECM is associated with non-invasive lesions. Upregulation of LAR-CSPG binding complexes results in strong binding of the tumor cells to the ECM, preventing cell invasion, and high levels of CSPGs elicit an astrocyte/microglia-mediated anti-invasion response. On the contrary, diffusely infiltrating tumor ECM lacks glycosylated CSPGs [28]. Interestingly, recent animal models suggest that temozolomide/dexamethasone combination therapy affects proteoglycan levels in the parenchymal ECM, potentially resulting in a proinvasive microenvironment [29].

Glioma cells also degrade the surrounding ECM to favor their migration. Proteases, among others, are the enzymes that tumor cells use to perform this activity. Matrix metalloproteinases, such as MMP-2 and MMP-9, are related to the tumor grade and the invasive capacity of glioma [30]. Other molecules involved in the degradation of the ECM are cysteine proteases, A disintegrin and metalloproteinases (ADAMs), and urokinase-type plasminogen activator (uPA). However, since low-grade gliomas with normal proteases levels are capable of invading the surrounding tissue, the role of proteases in the invasion of gliomas remains uncertain [31]. Nevertheless, in vitro assays show that a high migration capacity is associated with expression of MMP-2, MMP-9, uPA, and tissue plasminogen activator (tPA)[32].

### 2.3. Epithelial to Mesenchymal Transition

Epithelial to mesenchymal transition (EMT) is a biochemical process through which the cytoskeleton of polarized epithelial cells is remodelled, and they shift to a nonpolarized mesenchymal phenotype. Extensive evidence suggests that EMT is an essential process for tissue remodelling, wound repair and cancer metastasis. While in an epithelial state cells are held tightly and are anchored to the basement membrane, mesenchymal cells are mainly spindle-shaped and are loosely attached to the ECM through interaction with focal adhesion molecules. Specific transcription factors such as Snail and Slug, the zinc-finger E-box-binding homeobox (ZEB)1/2, and Twist1/2 are considered the main regulators of the EMT process, as they regulate transcription of genes, including N-cadherin, vimentin, and fibronectin that are typically expressed in mesenchymal cells [33]. These factors simultaneously suppress the expression of epithelial markers such as E-cadherin, claudins, occludins, and cytokeratins. Loss of E-cadherin, in turn, results in Wnt signaling and accumulation of *β*-catenin, which leads to increased transcription of genes that promote cell proliferation and invasion [34]. In GBM cell lines, it was shown that silencing SNAIL reduced invasion, migration and proliferation [35], and expression level of Slug correlated with tumor grade [36]. Additionally, ZEB1/ZEB2 expression correlated with invasiveness and decreased survival of GBM patients [37]. Furthermore, Twist1 and Twist 2, which typically regulate stemness, were found to be associated with the invasive properties of GBM cell lines as they regulate the expression of key EMT-regulating genes such as MMP2, Slug, and HGF [38].

It is important to note that the role of cadherin switch as a hallmark of EMT in carcinomas is not well established in GBM, as these tumors are not epithelial in nature. E-cadherin is expressed at very low levels in neural tissues and is found only in a small proportion of aggressive GBM cells. On the other hand, N-cadherin is absent in epithelial tumors before the initiation of EMT, while it is highly expressed in astrocytes and regulates cell polarity and migration, resulting in a less regulated cell movement [37]. It was also shown that expression of N-cadherin negatively correlated with GBM tumor cell invasiveness, and its overexpression* in vitro* reduced cell migration and restored cell polarity [3, 39]. In addition, several studies showed that radiation treatment or anti-angiogenic therapy of primary GBMs resulted in transition to a mesenchymal phenotype in the recurrent tumors [40, 41]. In fact, radio-resistant glioma cells display upregulated expression of genes involved in the EMT pathway [40, 42]. This is further supported by an* in vivo* study in xenograft mouse models of GBM, demonstrating that the gene expression profile of proneural GBM shifted towards a mesenchymal signature upon radiation treatment [41].

In addition to the master regulators, several cytokines play a role in EMT. In particular, Tumor Necrosis Factor-*α* (TNF*α*) activation through NF*κ*B is essential for EMT induction [43]. In addition, interleukins such as IL6 contribute to stimulation of EMT. Other signals that regulate the EMT and originate from the tumor include growth factors including HGF, EGF, and PDGF and these are thought to activate EMT-related transcription factors [44–46].

### 2.4. Cytoskeletal Remodelling and Cell Motility

Cytoskeletal remodelling is a key process in the formation of invadopodia and lamellipodia that are necessary for tumor cell motility [47]. Glioma cells typically show a mesenchymal pattern of migration and passage through extracellular spaces that are smaller than their own nuclei. Mechanistically, glioma cells become polarized and fibroblast-like, with characteristic leading and trailing edges on the opposite ends of the cell. This leads to the outward extension of the cell membrane at the leading edge (pseudopod), which is in contact with the ECM through ITGs localized on the cell membrane. ITGs interact with adaptor molecules and signaling proteins, activating signals inside the cell (phosphorylation/dephosphorylation via focal adhesion kinase, FAK) [48]. Subsequently, membrane-type MMPs are recruited at the focal contacts to degrade and restructure the ECM via the production of soluble matrix metalloproteases, including MMP-2 and MMP-9. Finally, the cells contract by the acto-myosin complex engagement, resulting in focal contact disassembly, integrin recycling, detachment of the trailing edge, and, ultimately, cell invasion [49, 50].

Other important factors that regulate acto-myosin complex engagement during EMT include RHO GTPases, among which RHOA promotes formation of actin stress fibres. RAC1 and CDC42, on the other hand, regulate the formation of lamellipodia and filopodia. Following the activation of GTPases, the RHO-associated kinase (ROCK) cooperates with the formin diaphanous 1 (DIA1) to enhance actin polymerization and also induces the phosphorylation of myosin light chain to promote acto-myosin contraction and activation of LIM kinase (LIMK)[51]. Once activated by RAC1 or CDC42, the p21-activated kinase 1 (PAK1) activates target proteins that are involved in cell spreading and motility [52]. In glioma cells, RHO GTPases including RHOA and RAC regulate cytoskeletal rearrangements resulting in ameboid and mesenchymal cell motility and have been shown to promote migration and growth of glioma cells* in vitro* and* ex vivo* [53]. Furthermore, it has been described that transmembrane ion cotransporters induce cell migration and EMT through downstream activation of RHOA and RAC pathways [54]. Besides, several pathways including Wnt, PI3K/Akt, and ODZ1 have been shown to be associated with RhoA to regulate cytoskeletal changes that allows migration [55–57].

It is important to note that glioma cell motility is not only influenced by the biochemical processes associated with the ECM but also by biophysical properties such as cell density and the rigidity and geometry of the ECM [58]. Ulrich et al. demonstrated that increased rigidity of the ECM in gliomas results in formation of stress fibres and focal adhesions that enable more rapid migration of the cells [59]. Another component of the tumor microenvironment that plays a role in cell invasion is blood vessels. Notably, glioma cells do not intravasate the vessels but instead associate with the vascular walls and migrate along the vessels. It has been shown that bradykinin is secreted by the brain endothelial cells and functions as a chemotactic signal for glioma cells through binding to its receptor (BR-2) on the glioma cell surface resulting in subsequent intracellular Ca2+ oscillations [60]. Changes in Ca2+ levels in turn, regulate cell motility through acto-myosin-mediated contraction, regulation of tubulin dynamics, and controlling the activation of focal adhesion kinases that mediate cell adhesion to substrates in the ECM [61]. Movement of glioma cells along the vascular walls in turn alters the organization of the brain vasculature where astrocyte endfeet are closely associated with endothelial cells through anchorage with basement membrane [62]. Migration of glioma cells leads to displacement of astrocytes endfeet via degradation of the basement membrane around the blood vessel environment. This results in disruption and breakdown of the blood-brain barrier (BBB) and alterations in blood vessel diameter [62]. This enables glioma cells to gain access to oxygen and nutrients from the bloodstream. In addition to the cytoskeletal rearrangement, regulation of cell volume by voltage-gated chloride and potassium channels is another mechanism that regulates glioma cell migration [63].

### 2.5. Cross-Talk with Host Cells and Immune Modulation

Tumor cells integrate with supportive stromal cells that are components of the tumor microenvironment. Stromal cells secrete growth factors and molecules that have the capacity to alter the milieu in which neoplastic cells proliferate. In fact, the microenvironment has been demonstrated to play key regulatory roles in response to therapy and tumor progression [64]. It has recently been shown that astrocytic and oligodendrogliotic gliomas share similar glial lineages and that difference in bulk expression profiles between these glial tumors is primarily driven by composition of the tumor microenvironment [65]. Alterations in local immune and vascular networks have been shown to facilitate tumor growth in GBM thereby highlighting the exciting opportunity for immunomodulatory therapies.

Nearly a third of GBM mass is composed of glioma-associated macrophages (GAMs). Due to the breakdown of the blood-brain barrier, these GAMs are derived primarily from bone-marrow derived cells and, to a lesser extent, from local resident inflammatory cells [66]. Macrophages either adopt a proinflammatory M1 phenotype or anti-inflammatory M2 phenotype. Glioma cells release chemo-attractans, such as monocyte chemo-attractant protein-1 (MCP-1), fractalkine (CX3CL1), glial cell–derived neurotrophic factor (GDNF), and colony stimulating factor-1 (CSF)-1) that recruit GAMs to tumor tissue [67]. CSF-1 plays a key role as it also promotes recruited macrophages to adopt M2 phenotype that contributes to tumor invasion. In fact, immunomodulation of CSF-1 signaling using a CSF-1R inhibitor has demonstrated to shift macrophages back to M1 phenotype with promising preclinical utility that requires further assessment [68].

Extensive body of literature suggests that GAMs are not simple passenger cells in the tumor microenvironment as they play a key role in regulating tumor growth and invasion with complex interactions with many other cell types [69, 70]. Importantly, GAMs secrete several factors with primary effects on tumor cells. For example, when exposed to glioma cells, GAMs upregulate expression of membrane type 1–matrix metalloproteinase (MT1-MMP) that cleaves pro-MMP2 to facilitate degradation of the extracellular matrix and GBM invasion. Moreover, GAMs secrete several oncogenic factors such as transforming growth factor beta (TGFß), which enhances glioma cell migration by upregulating integrin expression and contributes to the degradation of extra-cellular matrix components by inducing MMP2 expression and suppressing the expression of tissue inhibitor of metalloproteinases (TIMP)-2 [71, 72]. Although the interaction between neoplastic and stromal cells is complex, more thorough understanding of this crosstalk facilitates exploration of immune-modulatory compounds for GBM treatment.

### 2.6. Molecular Pathways in GBM Invasion

Large-scale genetic analyses have demonstrated that multiple signaling networks are employed by GBM cells to promote tumor growth and invasion. The most comprehensively studied pathways involved in GBM invasion include* PI3K/Akt*, Wnt/ß-catenin, Hedegehog, TGFß, and Tyrosine kinase receptors, which are involved in the activation of EMT-related cellular processes to promote tumor cell dissemination and invasion [73, 74]. Furthermore, as the structure of function of the ECM is critical for tumor cell invasion, dysfunction of ECM and its cognate receptor integrins may lead to aberrant activation of signaling pathways including Ras/Raf/MAPK, Raf/JNK, Rho/Rac/PAK, and PI3K/Akt/mTOR, which shape the tumor microenvironment and regulating tumor growth, angiogenesis, and invasion [75].

#### 2.6.1. Receptor Tyrosine Kinases

Many of the signal transduction pathways that regulate the tumor microenvironment, including Ras/Raf/MAPK, Raf/JNK, Rho/Rac/PAK, and PI3K/Akt/mTOR, are convergent downstream signaling pathways of RTKs, implicating their role in GBM invasiveness and aggressiveness [76]. Furthermore, as ECM serves as a reservoir for several growth factors including VEGF, EGF, PDGF, and TGF-*β*, secretion of these factors and their interaction with their receptors may lead to the activation of these signaling pathways, resulting in uncontrolled cell behaviors in tumor growth, angiogenesis, and invasion [77].

The Phosphoinositide-3-kinase (PI3K) signaling cascade is one of the main canonical pathways that have been implicated in GBM pathogenesis. This pathway transduces extracellular signals via receptor tyrosine kinases (RTKs) to regulate a series of biological processes such as cellular metabolism, growth, survival, and invasion. The PI3K pathway can be activated through interaction of ligands such as the epidermal growth factor (EGF) and TGFß with their respective RTKs. Induction of PI3K leads to activation of Akt family of kinases that regulate cell growth and survival. Regulation of the PI3K-Akt signaling pathway occurs through the tumor suppressor phosphatase and tensin homolog (PTEN) protein that dephosphorylates and, thereby, inactivates Akt [78].

Constitutional activation of the PI3K-Akt pathway is implicated in many cancers. In GBM, this pathway is activated by two frequent alterations, an in-frame deletion of amino acids 6–273 in EGFRvIII resulting in a mutant EGFR protein which is present in more than 50% of high grade gliomas and its activation is ligand-independent [79] and oncogenic mutations in PTEN detected in up to 40% of adult gliomas [80]. Both alterations result in increased expression of matrix metalloproteinases including MMP-2 and MMP9 that facilitate degradation of ECM and lead to tumor invasiveness [79]. The PI3K pathway is also activated by gain-of-function mutations in the PI3K catalytic subunit gene (PIK3CA). These mutations occur in up to 10% of GBMs and result in constitutive activation of the pathway with downstream effects similar to those promoted by* EGFRvIII *and* PTEN* mutations [81]. The key role of PI3K-Akt pathway in oncogenesis has sparked increasing interest in using small molecular inhibitors to target this pathway.

Additionally, the RTK c-Met and its ligand hepatocyte growth factor (HGF)/Scatter factor are overexpressed in gliomas and they have been shown to play a role in cell proliferation, invasion, angiogenesis and survival in several cancers [82]. EGFR and c-Met are known to trigger similar signal transduction pathways and their crosstalk in solid tumors affects the duration and strength of the response [83] and overall tumor malignancy. Notably, coexpression of EGFR and c-Met in GBM leads to deregulated EGFR signaling and increased HGF binding to c-Met, which in turn, promotes cell invasion [84].

In addition to EGFR and cMET, Wang et al. have demonstrated that the RTK Mer (MerTK) is overexpressed in GBM and this is accompanied with increased invasiveness [85]. Their results indicate that MerTK expression is maintained in primary GBM-derived tumour cells grown in stem cell cultures but is reduced significantly in serum-containing culture conditions, accompanied with downregulation of Nestin and Sox2. Furthermore, depletion of MerTK disrupts the round morphology of glioma cells and decreases their invasiveness. Additionally, the expression and phosphorylation of myosin light chain strongly correlated with activation of MerTK, suggesting that the effect of MerTK on glioma cell invasion is mediated by the ability of acto-myosin to contract. Importantly, DNA damage resulted in upregulation and phosphorylation of MerTK, protecting the cells from apoptosis. Collectively, RTKs appear as attractive therapeutic targets for the treatment of the malignant gliomas.

#### 2.6.2. Wnt (Canonical and ß-Catenin-Independent Pathways)

WNT signaling pathway is a crucial regulator of proliferation, migration and cell fate in the central nervous system during embryogenesis [86]. However, deregulation of this pathway also has oncogenic properties in mature cells. Abnormal WNT pathway activation is implicated in various cancers, including GBMs [87, 88]. Proteins of the WNT family bind to transmembrane Frizzled receptors [86] and downstream events can be divided into canonical ß-catenin-dependent and ß-catenin-independent pathways.

Activation of the canonical WNT pathway leads to disassembly of the transmembrane receptors of the ß-catenin destruction complex, consisting of the GSK3B, AXIN and adenomatous polyposis coli (APC) [86]. As a result, ß-catenin accumulates in the cytoplasm and translocates into the nucleus where it regulates TCF-LEF-dependent transcription. The classical targets of the canonical WNT pathway include cyclin D1 (CCND1), c-myc, COX2, and SOX2. Studies have demonstrated that the canonical pathway is important for glioma stem cell maintenance [89, 90]. In contrast, the ß-catenin independent pathway mainly regulates cell motility and polarity. This pathway is activated through WNT2, WNT4, WNT5A, WNT6, and WNT11 factors and leads to upregulation of the planar cell polarity (PCP) and calcium pathways [86].

In addition, WNT signaling is a major factor in epithelial-mesenchymal transition (EMT) and tumor invasion. Several studies have demonstrated that WNT pathway activation enhances the motility of cancer cells [87, 91]. Specifically, in GBMs constitutive activation of ß-catenin leads to increased tumor invasion, while inhibition of ß-catenin suppressed cell proliferation and invasion [87]. Furthermore, knockdown of WNT5A downregulated expression of MMP and suppressed glioma cell migration and invasion [91]. The building evidence of WNT pathway in GBM invasion provides a therapeutic rationale for targeting this pathway. Kahlert et al. found that the Wnt/*β*-catenin pathway is mainly activated within cells located at the invasive edge of the mesenchymal tumors. Furthermore, they found that this pathway mainly promotes tumor cell migration* in vitro *by inducing the expression of Zeb1, Twist1, and Slug [87].

#### 2.6.3. Hedegehog-GLI1

Similar to WNT pathway, the Hedgehog pathway plays a crucial role in the development of the central nervous system. Hedgehog pathway dysfunction during embryogenesis leads to congenital defects such as microcephaly or cyclopia. In many cancers including glioma, the Hedgehog pathway is upregulated and plays a role in tumorigenesis and tumor progression. Generally, Sonic hedgehog (SHH), Indian hedgehog (IHH), and Desert hedgehog (DHH) ligands can activate the Hedgehog pathway by binding to the transmembrane protein Patched (PTCH1). Hedgehog pathway activation leads to upregulation of GLI1, PTCH1, cyclin D2 (CCND2), Bcl-2, and VEGF. In addition, Hedgehog pathway modulates the expression of stemness genes, such as NANOG, OCT4, and SOX [92].

Although GLI1 amplification is relatively rare in GBMs, a novel truncated isoform, tGLI1, has been linked to increased cell motility and tumor invasion in GBM and breast cancer [93, 94]. This isoform is the result of alternative splicing and lacks exon 3 and part of exon 4. The tGLI1 isoform is undetectable in normal cells but expressed in GBM [93]. Furthermore, tGLI1 upregulates heparanase expression, which remodels the ECM and releases angiogenic factors [95]. The inhibition of hedgehog pathway with cyclopamine and RNA interference techniques inhibited glioma cell migration and tumor invasion [96, 97].

Epigenetic modulators may also play a role in hedgehog pathway activation. Bromodomain-containing protein 4 (BRD4) is a critical regulator of GLI1 transcription through direct occupancy of the gene promoter [98, 99]. In addition, lysine acetyltransferase 2B (KAT2B) is a positive cofactor in the Hedgehog pathway and depletion of KAT2B led to reduced expression of Hedgehog target genes [100]. Therefore, therapeutic strategies targeting the epigenetic modulators, such as BET-inhibitors and acetyltransferase inhibitors, are promising therapeutic options.

#### 2.6.4. Nuclear Factor-*κ*B

NF-*κ*B is a designation used for a family of highly regulated dimer transcription factors. They are usually elevated in GBM and contribute to the survival of migratory tumor cells [101]. Signaling pathways triggered by growth factor receptors, including EGFR and PDGFR, contribute to tumor development in GBM and NF-*κ*B plays key roles in these pathways [102, 103]. Among GBM subtypes, the mesenchymal phenotype is the most aggressive because it is highly invasive and radio-resistant [104] and associates with poor patient outcome. A transition of GBM cells from less aggressive phenotypes (i.e., proneural) to cells with mesenchymal features can be promoted by activation of NF*κ*B signaling [105]. Moreover, NF*κ*B activation in mesenchymal GBM cells mediates cell migration and tumor invasion through upregulation of NF*κ*B target genes, including cell chemoattractants (IL-8, MCP-1) and matrix metalloproteinases (MMP-9) [106]. This signaling pathway can be activated by a number of stimuli, including ECM components such as hyaluronic acid, through binding to TLR4, differentiation of GBM stem-like cells [27, 107], and cytokines that may be released by infiltrating monocytes/macrophages or surrounding parenchymal cells. To this end, when RANKL, a member of the TNF family, is upregulated in GBM cells, it activates neighbouring astrocytes through NF*κ*B signaling which leads to secretion of cytokines, such as TGFß, and promotes GBM cell invasion [108]. Thus, NF*κ*B-mediated invasiveness may occur when this signaling pathway is activated either in GBM cells or in cells in the tumor microenvironment.

## 3. The Clinical Implications of GBM Invasiveness

Invasiveness is one of the key features that allow GBM to overcome the current treatment strategies [109]. GBM initiating cells with enhanced invasive capacity have been identified in the peritumoral parenchyma. This cell subpopulation has a distinctive molecular profile [110, 111] and they are considered to be responsible for tumor recurrence, progression, and resistance to treatment [112, 113]. Furthermore, they could be involved in the gliomagenesis process [114].

Targeting tumor invasion and infiltration is a major clinical challenge. Novel pre- and intraoperative imaging techniques are being developed to accurately assess the extent of parenchymal infiltration in the clinical setting. Besides, new insights into potential therapeutic approaches have been recently reported.

### 3.1. Assessment of GBM Invasion in the Clinical Setting

#### 3.1.1. Imaging GBM Invasion

The radiological definition of infiltrated parenchyma remains unclear and the current imaging techniques, summarized in Table 1, are limited to accurately recognize the extent of tumor invasion. This is particularly relevant in focal therapies, such as surgical resection, radiotherapy, or local chemotherapeutic agents, to precisely define the peritumoral area that requires treatment in order to obtain significant responses.

GBM-induced T2/FLAIR hyperintensity in the MRI represents the area of peritumoral oedema and tumor-induced alterations in the parenchyma. It is a result of changes in the composition of the ECM and impairment of the blood-brain barrier in a process associated with the expression of endogenous tenascin-C [115].

It has been widely demonstrated that glioma cells infiltrate the peritumoral T2/FLAIR high signal region beyond the contrast enhancement on the preoperative MRI [116, 117]. The peritumoral invasion results in a gradient of the apparent diffusion coefficient (ADC) and in a higher relative Cerebral Blood Volume (rCBV), due to the peritumoral hyper-cellularity and the consequent increase in perfusion [118].

Nevertheless, the distinction of the diffuse nonenhancing tumor invasion from the peritumoral vasogenic oedema can be challenging in the clinical practice [119, 120]. Several alternative MRI-based methods have been described to overcome this limitation, including multi-parametric machine-learning [121] and DTI-based imaging analyses [122]. For instance, the distinction between oedema and tumor invasion is feasible by using quantitative MR methods [119] or by combining changes in the ADC value and the signal intensity on FLAIR images [120].

Moreover, considering GBM's diffuse infiltration, the burden of tumor cell invasion in the “normal” brain is not yet possible by using imaging techniques [123]. It is well known that invading tumor cell can be found as far as the contralateral hemisphere [124] and current imaging techniques are limited in fully assessing, at the microscopic level, the tumor cells invading the parenchyma beyond the limits of the T2/FLAIR abnormalities [125]. Besides, it has been suggested that GBM invasive margin can be identified by using a combination of DTI, perfusion, and spectroscopy [122, 126].

Radiomic analyses have focused on the invasion-related radio-phenotype applying quantitative volumetric to assess the correlation between specific radiological invasion features and IDH mutation status, outcome, or response to surgery [127, 128]. Besides, MRI-based mathematical models incorporating invasion features are capable to classify nodular and diffuse GBMs, two groups with different outcome and response to treatment [129]. Alternatively, MRI DWI-based models use the ADC value as a measure of cellular density predicting the spatial microscopic tumor growth dynamics and generating maps of cell diffusion and proliferation rates [130].

Other imaging methods, as Positron Emission Tomography (PET), have been used to assess the parenchymal response to tumor invasion [131] and, more recently, to assess the infiltrative tumor volume [132–134]. For instance, [18] fluorothymidine (FLT)-PET-CT, a proliferation marker, shows that the tumor infiltration can extend up to 24 mm beyond the MRI-based T2 abnormality volume and it was useful to distinguish between infiltrative tumor and peritumoral oedema [132]. Similar results have been described by using other PET amino acid markers as Fluoroethylthyrosine [133], Tryptophan [134], and methionine [135].

#### 3.1.2. Intraoperative Identification of GBM Invasion

Intraoperatively, the tumor infiltrating the adjacent parenchyma maintains the macroscopic aspect of normal or oedematous brain parenchyma. Therefore, it is critical to develop and validate methods to accurately define the boundaries of the infiltrative tumor.

In the last two decades, the 5-aminolevulinic acid (5-ALA), an intermediate metabolite in the porphyrin intracellular pathway that results in the accumulation of fluorescent protoporphyrin IX molecule inside tumor cells, has been used to intraoperatively define the infiltrative tumor [136, 137]. Although 5-ALA fluorescence represents contrast-enhanced tumor in the MRI, an accurate correlation with T2/FLAIR changes remains unclear. It is widely accepted that 5-ALA fluorescence depicts more accurately the tumor burden than gadolinium; however its capacity to identify the infiltrative tumor is not fully understood due to a low negative predictive value [138, 139].

Moreover, the concordance between 5-ALA fluorescence and intraoperative MRI (iMRI) findings is still poorly understood. For instance, residual contrast enhancement in the iMRI after 5-ALA fluorescence-guided resection can be found in the majority of cases. Histopathological analysis of these regions revealed tumor core or tumor infiltration in 39 and 25% of cases, respectively [140, 141]. In other histopathological correlation studies, 5-ALA predicted tumor in strong and weak fluorescence regions. However, tumor tissue was still observed in fluorescence-negative regions in approximately half of the cases [142]. Besides, although the use of iMRI and 5-ALA fluorescence-guided surgery may increase the extent of resection, a significant impact in survival has not been established [143].

On the other hand, preoperative 18F-fluoroethyl-L-tyrosine (FET)-PET can predict 5-ALA fluorescence [144]. However, more recent analyses have shown contradictory results. Roessler at al. described that 5-ALA had higher sensitivity than 18F-FET-PET to detect the infiltrative tumor surrounding the contrast-enhanced region [145]. On the contrary, Floeth et al. concluded that 18F-FET PET is more sensitive to detect glioma tissue than 5-ALA fluorescence [146]. Further research is needed to fully understand the correlation between both techniques.

Fluorescein sodium (Fl-Na) is another marker used in fluorescence-guided surgery. Despite a good correlation of Fl-Na and histopathological [147], 5-ALA has demonstrated to be superior in identifying tumor cells in the peri-tumoral area beyond the contrast-enhanced tumor when compared to Fl-Na. While Fl-NA accumulation is associated with blood-brain barrier disruption, 5-ALA is mainly dependent on the protoporphyrin tumor cell pathway [148].

Intraoperative ultrasound (US) is another intraoperative resource used to assess tumor extension [149, 150]. In brightness mode (B-Mode) GBM appears as a heterogeneous echogenic mass with hyperechogenic boundaries and, in the majority of LGG, the B-mode hyperechogenicity overlaps with the preoperative T2/FLAIR MRI hyper-intensity [150–152]. Nevertheless, in both cases, the distinction between infiltrative tumor and associated oedema can be challenging, especially in advanced stages of the resection when surgery-related oedema and other artefacts may interfere with the US imaging [153]. Although intraoperative US is a promising tool to assess the infiltrative tumor, a better understanding of the underlying mechanisms is needed along with the development of multimodal intraoperative US imaging approaches integrating contrast-enhanced ultrasound [152, 154] and elastosonography [151].

Among other techniques described to identify the boundaries of the infiltrative GBM during the surgical resection, intraoperative confocal microscopy is an emerging approach capable of identifying fluorescein-, indocyanine green-, or acriflavine hydrochloride-enhanced differences in cell density and cellular morphology corresponding with the T2 hyper-intensity on MR imaging [117, 155–157]. Furthermore, this technique can potentially identify the tumor margins at a microscopic level and distinguish them from perilesional parenchyma [155].

Finally, optical coherence tomography, a real-time tissue microstructure imaging technique based on low-coherence interferometry in the near infra-red range of wavelengths, is another promising tool for assessing the tumor infiltrative margin in gliomas. It provides comprehensive qualitative and quantitative analysis of the tumor and the peritumoral tissue, generating color-coded maps that correlate with the histological findings and help to accurately identify the tumor boundaries [158–161].

### 3.2. Therapeutic Approaches Targeting GBM Invasion

The current standard of care for patients with GBM involves surgical resection and adjuvant chemo-radiation with temozolomide [1]. It is widely accepted that the infiltrated parenchyma is associated with recurrence and resistance to treatment, thereby playing a central role in each step of the treatment [162].

#### 3.2.1. Surgical Resection of the Infiltrative Tumor

In GBM, tumor cell invasiveness can lead to the infiltration or destruction of surrounding parenchyma resulting in neurological deficits [63, 163]. It has been proven that gross total resection of the contrast-enhanced tumor improves overall outcome [164, 165]. However, this approach might disregard the tumor burden invading the surrounding parenchyma, which could be potentially resected if eloquent areas are not compromised [166].

Thus far, several studies have shown that resection of the infiltrative portion of the tumor, based on DTI, ADC, or T2/FLAIR abnormalities is associated with longer progression-free survival (PFS) and overall survival (OS) [166–170]. However, a recent analysis of 245 primary GBMs did not find a significant difference in recurrence and survival associated with the postoperative FLAIR volume [171].

Although there is evidence supporting that resection of the infiltrative tumor can result in better outcomes, opposite results highlight the need for further research, as it remains unclear the more appropriate method to identify the areas of the surrounding parenchyma with greater tumor cell density and to distinguish them from the oedematous brain [120].

#### 3.2.2. Radiation Therapy Targeting GBM Invasiveness

Accurate tumor volume definition is critical in conformal or intensity-modulated radiotherapy (IMRT) planning. Analogously to surgical approaches, a subtherapeutic radiation dose within the tumor may result in treatment failure and recurrence, whereas whole-brain dose increments may lead to radiation-induced toxicity [133]. Moreover, a sublethal irradiation dose may enhance invasion in GBM [172, 173]. Another suggested mechanism of tumor recurrence is the proinvasive ECM remodelling in the tumor microenvironment in response to ionizing radiation [25].

Despite the infiltrative nature of GBM, radiation planning protocols have evolved from whole brain radiotherapy towards more tailored tumor volume targets, partially based on that the great majority of recurrences arise within 2 cm from the primary site [174, 175]. In this context, it remains unclear if targeting the MRI-defined infiltrative tumor results in better PFS and OS. Moreover, in clinical practice there is a considerable variation in target volume definition without significant differences in outcome, from using a 2-3 cm margin on the T1 contrast-enhanced tumor to a 2 cm margin on the T2/FLAIR hyper-intensity, as recommended by the European Organization for Research and Treatment of Cancer or the Radiation Therapy Oncology Group, respectively [176, 177]. In fact, by targeting the tumor area with a margin of 2 cm and without using the peritumoral oedema as tumor volume, Chang et al. achieved similar recurrence pattern results [175]. Further research is needed to assess whether this is a result of the overall lack of benefit from radiation therapy or if targeting the infiltrative tumor burden with radiation does not significantly impact the outcome [177].

On the other hand, the use of DTI-based clinical target volumes (CTV) has been proposed, as they are smaller than the ones based on the T2-hyperintensity, sparing the peritumoral oedema. Besides, this reduction in the CTVs could allow dose escalation [178, 179]. Furthermore, approaches taking into account tumor growth dynamics have been developed, by defining the CTVs based on DTI-derived mathematical growth models. Although this approach could be more effective at targeting cancer cells and preserving healthy tissue, further research is warranted to assess its outcome and tumor recurrence [123, 180]

Other approaches for CTV definition are based on PET findings. For instance, a higher dose coverage of 18F-FET-PET tumor regions is positively correlated with time to progression and PET-based CTVs better-predicted failure sites when compared to MRI-based CTVs [133, 181], although current ongoing protocols are trying to better define the impact of PET-based tumor delineation in outcome [182].

#### 3.2.3. Therapeutic Targets in GBM Invasion

Overall, current commonly used therapies for GBM, including alkylating agents as Temozolomide (TMZ) and the anti-VEGF compound Bevacizumab, failed in targeting glioma cell invasion. Although TMZ can potentially inhibit invasion in vitro [183], this effect is not significant in the clinical practice and several resistance mechanisms to alkylating agents have been proposed [184]. Among them, the lack of blood-brain permeability in T2/FLAIR hyperintensity areas [185, 186] and the resistance mechanisms intrinsic to GSC in the infiltrative tumor are intimately associated with the GBM invasive capacity [112, 187]. On the other hand, Bevacizumab could lead to a hypoxic environment resulting in enhanced glioma cell invasion of the normal parenchyma [188, 189].

Considering the lack of an effective therapeutic approach against GBM invasiveness, further research is warranted to better understand the invasion pathways contributing to glioma cell infiltration and, consequently, to develop new therapeutic agents. An effective therapeutic strategy should target both infiltrative GBM cells and the tumor cell-stroma interaction [190].

Up to now, no clinically transferable results have been achieved after trying to target some of the mechanisms involved in GBM invasion, including cytoskeleton reorganization and cell motility, cell adhesion, and degradation of ECM [57, 162]

Current areas of research include several potential targets in glioma cell invasion pathways. Glutamate-mediated infiltration inhibition has been assessed in several Phases I-II trials with promising results. Besides, the role of different tumor cell ion channels and transporters, microtubule-based tumor cell network, microRNA-related invasion, and the mechanisms involved in the interaction between the tumor and the host open potential opportunities for targeted therapy approaches [109, 162, 190]

## 4. Conclusion

**Figure 1:**
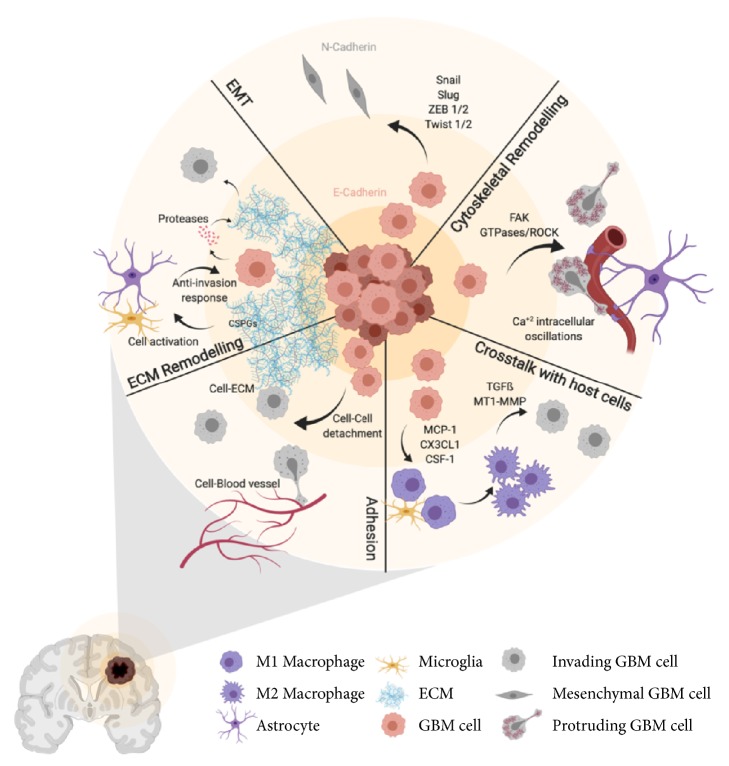
*Cellular processes involved in GBM cell invasion*. Schematic summary of the processes involved in the invasive capacity of GBM cells including cell-to-cell and cell-to-ECM adhesion, ECM remodelling, EMT, cytoskeletal remodelling, and cross-talk with host cells. See text for details (created with Biorender.com).

The GBM invasiveness capacity is one of the main features contributing to tumor recurrence, treatment resistance, and low survival rates. It results from an intricate combination of several signalling routes, mainly receptor tyrosine kinases and transcriptional pathways and also cellular processes that include cytoskeletal remodelling and interactions with ECM components and host cells (Figure 1). Although significant advances have been made in the last decade, the complexity of this protein interaction network and the lack of understanding about the contribution of each one of these mechanisms to glioma cell invasiveness have hampered the translation of novel therapeutic strategies into the clinic. Further research integrating key elements in the process of invasion will be needed to unravel efficient combination therapies to avoid tumor progression. Novel preoperative and intraoperative imaging techniques have been recently developed to help the clinician to recognize and treat the infiltrative portion of the GBM. Nevertheless, this portion of the tumor remains elusive to these methods. Therefore, improvement in revealing the presence of invasive tumor cells would be needed in the clinical practice to significantly impact the prognosis of patients with GBM.

## Figures and Tables

**Table 1:** Pre- and intraoperative methods to assess GBM's invasive capacity.

Preoperative methods	Intraoperative methods
*MRI-based*	*Fluorescence-guidance*
T2/FLAIR hyperintensity	5-aminolevulinic acid (5-ALA)
DTI	Fluorescein sodium (Fl-Na)
DWI (ADC and FA)	*iMRI-based T2/FLAIR*
Perfusion	*Intraoperative Ultrasound*
Spectroscopy	Contrast enhanced US
Quantitative MR	Elastosonography
Radiomics radiophenotype	*Intraoperative confocal microscopy *
*PET-based*	Fluorescein
Fluorothymidine	Indocyanine green
Fluoroethylthyrosine	Acriflavine hydrochloride
Tryptophan	*Optical coherence tomography*
Methionine	

MRI= Magnetic Resonance Imaging, FLAIR= fluid attenuated inversion recovery, DTI= Diffusion tensor imaging, PET= Positron emission tomography, and iMRI= intraoperative MRI.

## References

[1] Stupp R., Mason W. P., van den Bent M. J. (2005). Radiotherapy plus concomitant and adjuvant temozolomide for glioblastoma. *The New England Journal of Medicine*.

[2] Soffietti R., Baumert B. G., Bello L. (2010). Guidelines on management of low-grade gliomas: report of an EFNS-EANO Task Force. *European Journal of Neurology*.

[3] Asano K., Duntsch C. D., Zhou Q. (2004). Correlation of N-cadherin expression in high grade gliomas with tissue invasion. *Journal of Neuro-Oncology*.

[4] Claes A., Idema A. J., Wesseling P. (2007). Diffuse glioma growth: a guerilla war. *Acta Neuropathologica*.

[5] Duenisch P., Reichart R., Mueller U. (2011). Neural cell adhesion molecule isoform 140 declines with rise of WHO grade in human gliomas and serves as indicator for the invasion zone of multiform glioblastomas and brain metastases. *Journal of Cancer Research and Clinical Oncology*.

[6] Jayaram S., Balakrishnan L., Singh M. (2018). Identification of a Novel Splice Variant of Neural Cell Adhesion Molecule in Glioblastoma Through Proteogenomics Analysis. *OMICS: A Journal of Integrative Biology*.

[7] Yu J. A., Sadaria M. R., Meng X. (2012). Lung cancer cell invasion and expression of intercellular adhesion molecule-1 (ICAM-1) are attenuated by secretory phospholipase A_2_ inhibition. *The Journal of Thoracic and Cardiovascular Surgery*.

[8] Frank P. G., Lisanti M. P. (2008). ICAM-1: role in inflammation and in the regulation of vascular permeability. *American Journal of Physiology-Heart and Circulatory Physiology*.

[9] Lin Y., Shun C., Wu M., Chen C. (2006). A novel anticancer effect of thalidomide: inhibition of intercellular adhesion molecule-1-mediated cell invasion and metastasis through suppression of nuclear factor- B. *Clinical Cancer Research*.

[10] Kesanakurti D., Chetty C., Rajasekhar Maddirela D., Gujrati M., Rao J. S. (2013). Essential role of cooperative NF-*κ*B and Stat3 recruitment to ICAM-1 intronic consensus elements in the regulation of radiation-induced invasion and migration in glioma. *Oncogene*.

[11] Lin J., Tsai J., Chao T., Ma H., Liu W. (2019). Musashi-1 enhances glioblastoma migration by promoting ICAM1 translation. *Neoplasia*.

[12] Takada Y., Ye X., Simon S. (2007). The integrins. *Genome Biology*.

[13] Paolillo M., Serra M., Schinelli S. (2016). Integrins in glioblastoma: Still an attractive target?. *Pharmacological Research*.

[14] Desgrosellier J. S., Cheresh D. A. (2010). Integrins in cancer: biological implications and therapeutic opportunities. *Nature Reviews Cancer*.

[15] Renner G., Noulet F., Mercier M.-C. (2016). Expression/activation of *α*5*β*1 integrin is linked to the *β*-catenin signaling pathway to drive migration in glioma cells. *Oncotarget *.

[16] Serres E., Debarbieux F., Stanchi F. (2014). Fibronectin expression in glioblastomas promotes cell cohesion, collective invasion of basement membrane in vitro and orthotopic tumor growth in mice. *Oncogene*.

[17] Mikheev A. M., Mikheeva S. A., Trister A. D. (2015). Periostin is a novel therapeutic target that predicts and regulates glioma malignancy. *Neuro-Oncology*.

[18] Ding Q., Stewart J. J., Prince C. W. (2002). Promotion of malignant astrocytoma cell migration by osteopontin expressed in the normal brain: differences in integrin signaling during cell adhesion to osteopontin versus vitronectin. *Cancer Research*.

[19] Hehlgans S., Haase M., Cordes N. (2007). Signalling via integrins: Implications for cell survival and anticancer strategies. *Biochimica et Biophysica Acta (BBA) - Reviews on Cancer*.

[20] Lawson C. D., Burridge K. (2014). The on-off relationship of Rho and Rac during integrin-mediated adhesion and cell migration. *Small GTPases*.

[21] Scaringi C., Minniti G., Caporello P., Enrici R. M. (2012). Integrin inhibitor cilengitide for the treatment of glioblastoma: A brief overview of current clinical results. *Anticancer Reseach*.

[22] Delamarre E., Taboubi S., Mathieu S. (2009). Expression of integrin alpha6beta1 enhances tumorigenesis in glioma cells. *The American Journal of Pathology*.

[23] Herrera-Perez M., Voytik-Harbin S. L., Rickus J. L. (2015). Extracellular matrix properties regulate the migratory response of glioblastoma stem cells in three-dimensional culture. *Tissue Engineering Part A*.

[24] de Gooijer M. C., Guillén Navarro M., Bernards R., Wurdinger T., van Tellingen O. (2018). An experimenter’s guide to glioblastoma invasion pathways. *Trends in Molecular Medicine*.

[25] Yoo K., Suh Y., An Y. (2018). Proinvasive extracellular matrix remodeling in tumor microenvironment in response to radiation. *Oncogene*.

[26] Chen J. E., Lumibao J., Blazek A., Gaskins H. R., Harley B. (2018). Hypoxia activates enhanced invasive potential and endogenous hyaluronic acid production by glioblastoma cells. *Biomaterials Science*.

[27] Ferrandez E., Gutierrez O., Segundo D. S., Fernandez-Luna J. L. (2018). NF*κ*B activation in differentiating glioblastoma stem-like cells is promoted by hyaluronic acid signaling through TLR4. *Scientific Reports*.

[28] Kim Y., Kang H., Powathil G. (2018). Role of extracellular matrix and microenvironment in regulation of tumor growth and LAR-mediated invasion in glioblastoma. *PLoS ONE*.

[29] Tsidulko A. Y., Bezier C., de La Bourdonnaye G. (2018). Conventional anti-glioblastoma chemotherapy affects proteoglycan composition of brain extracellular matrix in rat experimental model in vivo. *Frontiers in Pharmacology*.

[30] Wang M., Wang T., Liu S., Yoshida D., Teramoto A. (2003). The expression of matrix metalloproteinase-2 and -9 in human gliomas of different pathological grades. *Brain Tumor Pathology*.

[31] Lakka S. S., Gondi C. S., Yanamandra N. (2004). Inhibition of cathepsin B and MMP-9 gene expression in glioblastoma cell line via RNA interference reduces tumor cell invasion, tumor growth and angiogenesis. *Oncogene*.

[32] Kaphle P., Li Y., Yao L. (2019). The mechanical and pharmacological regulation of glioblastoma cell migration in 3D matrices. *Journal of Cellular Physiology*.

[33] Kahlert U. D., Joseph J. V., Kruyt F. A. (2017). EMT- and MET-related processes in nonepithelial tumors: importance for disease progression, prognosis, and therapeutic opportunities. *Molecular Oncology*.

[34] McCrea P. D., Gottardi C. J. (2016). Beyond *β*-catenin: prospects for a larger catenin network in the nucleus. *Nature Reviews Molecular Cell Biology*.

[35] Myung J. K., Choi S. A., Kim S.-K., Wang K.-C., Park S.-H. (2014). Snail plays an oncogenic role in glioblastoma by promoting epithelial mesenchymal transition. *International Journal of Clinical and Experimental Pathology*.

[36] Yang H. W., Menon L. G., Black P. M., Carroll R. S., Johnson M. D. (2010). SNAI2/Slug promotes growth and invasion in human gliomas. *BMC Cancer*.

[37] Siebzehnrubl F. A., Silver D. J., Tugertimur B. (2013). The ZEB1 pathway links glioblastoma initiation, invasion and chemoresistance. *EMBO Molecular Medicine*.

[38] Mikheeva S. A., Mikheev A. M., Petit A. (2010). TWIST1 promotes invasion through mesenchymal change in human glioblastoma. *Molecular Cancer*.

[39] Camand E., Peglion F., Osmani N., Sanson M., Etienne-Manneville S. (2012). N-cadherin expression level modulates integrin-mediated polarity and strongly impacts on the speed and directionality of glial cell migration. *Journal of Cell Science*.

[40] Mahabir R., Tanino M., Elmansuri A. (2014). Sustained elevation of Snail promotes glial-mesenchymal transition after irradiation in malignant glioma. *Neuro-Oncology*.

[41] Halliday J., Helmy K., Pattwell S. S. (2014). In vivo radiation response of proneural glioma characterized by protective p53 transcriptional program and proneural-mesenchymal shift. *Proceedings of the National Acadamy of Sciences of the United States of America*.

[42] Kim Y., Yoo K., Cui Y. (2014). Radiation promotes malignant progression of glioma cells through HIF-1alpha stabilization. *Cancer Letters*.

[43] Storci G., Sansone P., Mari S. (2010). TNFalpha up-regulates SLUG via the NF-kappaB/HIF1alpha axis, which imparts breast cancer cells with a stem cell-like phenotype. *Journal of Cellular Physiology*.

[44] Kim J., Kong J., Chang H., Kim H., Kim A. (2016). EGF induces epithelial-mesenchymal transition through phospho-Smad2/3-Snail signaling pathway in breast cancer cells. *Oncotarget *.

[45] Liu F., Song S., Yi Z. (2017). HGF induces EMT in non-small-cell lung cancer through the hBVR pathway. *European Journal of Pharmacology*.

[46] Wu Q., Hou X., Xia J. (2013). Emerging roles of PDGF-D in EMT progression during tumorigenesis. *Cancer Treatment Reviews*.

[47] Stylli S. S., Kaye A. H., Lock P. (2008). Invadopodia: At the cutting edge of tumour invasion. *Journal of Clinical Neuroscience*.

[48] Hynes R. O. (2002). Integrins: bidirectional, allosteric signaling machines. *Cell*.

[49] Tam E. M., Wu Y. I., Butler G. S., Stack M. S., Overall C. M. (2002). Collagen binding properties of the membrane type-1 matrix metalloproteinase (MT1-MMP) hemopexin C domain. *The Journal of Biological Chemistry*.

[50] Wear M. A., Schafer D. A., Cooper J. A. (2000). Actin dynamics: Assembly and disassembly of actin networks. *Current Biology*.

[51] Narumiya S., Tanji M., Ishizaki T. (2009). Rho signaling, ROCK and mDia1, in transformation, metastasis and invasion. *Cancer and Metastasis Reviews*.

[52] Whale A., Hashim F. N., Fram S., Jones G. E., Wells C. M. (2011). Signaling to cancer cell invasion through PAK family kinases. *Front Biosci (Landmark Ed)*.

[53] Wang H., Han M., Whetsell W. (2014). Tax-interacting protein 1 coordinates the spatiotemporal activation of Rho GTPases and regulates the infiltrative growth of human glioblastoma. *Oncogene*.

[54] Ma H., Li T., Tao Z. (2019). NKCC1 promotes EMT-like process in GBM via RhoA and Rac1 signaling pathways. *Journal of Cellular Physiology*.

[55] Liu G., Yan T., Li X. (2018). Daam1 activates RhoA to regulate Wnt5ainduced glioblastoma cell invasion. *Oncology Reports*.

[56] Talamillo A., Grande L., Ruiz-Ontañon P. (2016). ODZ1 allows glioblastoma to sustain invasiveness through a Myc-dependent transcriptional upregulation of RhoA. *Oncogene*.

[57] Drappatz J., Norden A. D., Wen P. Y. (2014). Therapeutic strategies for inhibiting invasion in glioblastoma. *Expert Review of Neurotherapeutics*.

[58] Discher D. E., Janmey P., Wang Y. L. (2005). Tissue cells feel and respond to the stiffness of their substrate. *Science*.

[59] Ulrich T. A., de Juan Pardo E. M., Kumar S. (2009). The Mechanical Rigidity of the Extracellular Matrix Regulates the Structure, Motility, and Proliferation of Glioma Cells. *Cancer Research*.

[60] Montana V., Sontheimer H. (2011). Bradykinin Promotes the Chemotactic Invasion of Primary Brain Tumors. *The Journal of Neuroscience*.

[61] Martini F. J., Valdeolmillos M. (2010). Actomyosin Contraction at the Cell Rear Drives Nuclear Translocation in Migrating Cortical Interneurons. *The Journal of Neuroscience*.

[62] Watkins S., Robel S., Kimbrough I. F., Robert S. M., Ellis-Davies G., Sontheimer H. (2014). Disruption of astrocyte–vascular coupling and the blood–brain barrier by invading glioma cells. *Nature Communications*.

[63] Cuddapah V. A., Robel S., Watkins S., Sontheimer H. (2014). A neurocentric perspective on glioma invasion. *Nature Reviews Neuroscience*.

[64] Hirata E., Sahai E. (2017). Tumor Microenvironment and Differential Responses to Therapy. *Cold Spring Harbor Perspectives in Medicine*.

[65] Venteicher A. S., Tirosh I., Hebert C. (2017). Decoupling genetics, lineages, and microenvironment in IDH-mutant gliomas by single-cell RNA-seq. *Science*.

[66] De Palma M. (2016). Origins of brain tumor macrophages. *Cancer Cell*.

[67] Hambardzumyan D., Gutmann D. H., Kettenmann H. (2016). The role of microglia and macrophages in glioma maintenance and progression. *Nature Neuroscience*.

[68] Pyonteck S. M., Akkari L., Schuhmacher A. J. (2013). CSF-1R inhibition alters macrophage polarization and blocks glioma progression. *Nature Medicine*.

[69] Biswas S. K., Mantovani A. (2010). Macrophage plasticity and interaction with lymphocyte subsets: cancer as a paradigm. *Nature Immunology*.

[70] Hu F., a Dzaye O. D., Hahn A. (2015). Glioma-derived versican promotes tumor expansion via glioma-associated microglial/macrophages Toll-like receptor 2 signaling. *Neuro-Oncology*.

[71] Markovic D. S., Vinnakota K., Chirasani S. (2009). Gliomas induce and exploit microglial MT1-MMP expression for tumor expansion. *Proceedings of the National Acadamy of Sciences of the United States of America*.

[72] Wesolowska A., Kwiatkowska A., Slomnicki L. (2008). Microglia-derived TGF-*β* as an important regulator of glioblastoma invasion—an inhibition of TGF-*β*-dependent effects by shRNA against human TGF-*β* type II receptor. *Oncogene*.

[73] Singh S. K., Hawkins C., Clarke I. D. (2004). Identification of human brain tumour initiating cells. *Nature*.

[74] Hanahan D., Weinberg R. A. (2011). Hallmarks of cancer: the next generation. *Cell*.

[75] Manini I., Caponnetto F., Bartolini A. (2018). Role of microenvironment in glioma invasion: what we learned from in vitro models. *International Journal of Molecular Sciences*.

[76] Streulli C. H., Akhtar N. (2009). Signal co-operation between integrins and other receptor systems. *Biochemical Journal*.

[77] Plotnikov S. V., Pasapera A. M., Sabass B., Waterman C. M. (2012). Force fluctuations within focal adhesions mediate ECM-rigidity sensing to guide directed cell migration. *Cell*.

[78] Liu C., Wu H., Li Y. (2017). SALL4 suppresses PTEN expression to promote glioma cell proliferation via PI3K/AKT signaling pathway. *Journal of Neuro-Oncology*.

[79] Lal A., Glazer C. A., Martinson H. M. (2002). Mutant epidermal growth factor receptor up-regulates molecular effectors of tumor invasion. *Cancer Research*.

[80] Brennan C. W., Verhaak R. G., McKenna A. (2013). The somatic genomic landscape of glioblastoma. *Cell*.

[81] McNeill R. S., Stroobant E. E., Smithberger E. (2018). PIK3CA missense mutations promote glioblastoma pathogenesis, but do not enhance targeted PI3K inhibition. *PLoS ONE*.

[82] Gentile A., Trusolino L., Comoglio P. M. (2008). The Met tyrosine kinase receptor in development and cancer. *Cancer and Metastasis Reviews*.

[83] Trusolino L., Bertotti A., Comoglio P. M. (2010). MET signalling: principles and functions in development, organ regeneration and cancer. *Nature Reviews Molecular Cell Biology*.

[84] Velpula K. K., Dasari V. R., Asuthkar S., Gorantla B., Tsung A. J. (2012). EGFR and c-Met Cross Talk in Glioblastoma and Its Regulation by Human Cord Blood Stem Cells. *Translational Oncology*.

[85] Wang Y., Moncayo G., Morin P. (2013). Mer receptor tyrosine kinase promotes invasion and survival in glioblastoma multiforme. *Oncogene*.

[86] Dijksterhuis J. P., Petersen J., Schulte G. (2014). WNT/Frizzled signalling: Receptor-ligand selectivity with focus on FZD-G protein signalling and its physiological relevance: IUPHAR Review 3. *British Journal of Pharmacology*.

[87] Kahlert U. D., Maciaczyk D., Doostkam S. (2012). Activation of canonical WNT/*β*-catenin signaling enhances in vitro motility of glioblastoma cells by activation of ZEB1 and other activators of epithelial-to-mesenchymal transition. *Cancer Letters*.

[88] Cui C., Zhou X., Zhang W., Qu Y., Ke X. (2018). Is *β*-catenin a druggable target for cancer therapy?. *Trends in Biochemical Sciences*.

[89] Bhuvanalakshmi G., Gamit N., Patil M. (2018). Stemness, pluripotentiality, and Wnt antagonism: sFRP4, a Wnt antagonist mediates pluripotency and stemness in glioblastoma. *Cancers*.

[90] Wang G., Shen J., Sun J. (2017). Cyclophilin a maintains glioma-initiating cell stemness by regulating Wnt/*β*-catenin signaling. *Clinical Cancer Research*.

[91] Kamino M., Kishida M., Kibe T. (2011). Wnt-5a signaling is correlated with infiltrative activity in human glioma by inducing cellular migration and MMP-2. *Cancer Science*.

[92] Carpenter R. L., Lo H. (2012). Identification, functional characterization, and pathobiological significance of GLI1 isoforms in human cancers. *Vitamins and Hormones*.

[93] Rimkus T. K., Carpenter R. L., Sirkisoon S. (2018). Truncated glioma-associated oncogene homolog 1 (tGLI1) mediates mesenchymal glioblastoma via transcriptional activation of CD44. *Cancer Research*.

[94] Sirkisoon S. R., Carpenter R. L., Rimkus T. (2018). Interaction between STAT3 and GLI1/tGLI1 oncogenic transcription factors promotes the aggressiveness of triple-negative breast cancers and HER2-enriched breast cancer. *Oncogene*.

[95] Carpenter R. L., Paw I., Zhu H. (2015). The gain-of-function GLI1 transcription factor TGLI1 enhances expression of VEGF-C and TEM7 to promote glioblastoma angiogenesis. *Oncotarget*.

[96] Wang K., Pan L., Che X., Cui D., Li C. (2010). Sonic Hedgehog/GLI1 signaling pathway inhibition restricts cell migration and invasion in human gliomas. *Neurological Research*.

[97] Uchida H., Arita K., Yunoue S. (2011). Role of sonic hedgehog signaling in migration of cell lines established from CD133-positive malignant glioma cells. *Journal of Neuro-Oncology*.

[98] Wang Y., Sui X., Sui Y. (2015). BRD4 induces cell migration and invasion in HCC cells through MMP-2 and MMP-9 activation mediated by the Sonic hedgehog signaling pathway. *Oncology Letters*.

[99] Tang Y., Gholamin S., Schubert S. (2014). Epigenetic targeting of Hedgehog pathway transcriptional output through BET bromodomain inhibition. *Nature Medicine*.

[100] Malatesta M., Steinhauer C., Mohammad F., Pandey D. P., Squatrito M., Helin K. (2013). Histone Acetyltransferase PCAF Is Required for Hedgehog-Gli-Dependent Transcription and Cancer Cell Proliferation. *Cancer Research*.

[101] Smith D., Shimamura T., Barbera S., Bejcek B. E. (2008). NF-*κ*B controls growth of glioblastomas/astrocytomas. *Molecular and Cellular Biochemistry*.

[102] Shih A. H., Holland E. C. (2006). Platelet-derived growth factor (PDGF) and glial tumorigenesis. *Cancer Letters*.

[103] Bonavia R., Inda M. M., Vandenberg S. (2012). EGFRvIII promotes glioma angiogenesis and growth through the NF-*κ*B, interleukin-8 pathway. *Oncogene*.

[104] Carro M. S., Lim W. K., Alvarez M. J. (2010). The transcriptional network for mesenchymal transformation of brain tumours. *Nature*.

[105] Bhat K. P. L., Balasubramaniyan V., Vaillant B. (2013). Mesenchymal differentiation mediated by NF-*κ*B promotes radiation resistance in glioblastoma. *Cancer Cell*.

[106] Tchoghandjian A., Jennewein C., Eckhardt I., Rajalingam K., Fulda S. (2013). Identification of non-canonical NF-*κ*B signaling as a critical mediator of Smac mimetic-stimulated migration and invasion of glioblastoma cells. *Cell Death & Disease*.

[107] Nogueira L., Ruiz-Ontañon P., Vazquez-Barquero A. (2011). Blockade of the NF*κ*B pathway drives differentiating glioblastoma-initiating cells into senescence both in vitro and in vivo. *Oncogene*.

[108] Kim J., Jin X., Sohn Y. (2014). Tumoral RANKL activates astrocytes that promote glioma cell invasion through cytokine signaling. *Cancer Letters*.

[109] Vehlow A., Cordes N. (2013). Invasion as target for therapy of glioblastoma multiforme. *Biochimica et Biophysica Acta (BBA)—Reviews on Cancer*.

[110] Gill B. J., Pisapia D. J., Malone H. R. (2014). MRI-localized biopsies reveal subtype-specific differences in molecular and cellular composition at the margins of glioblastoma. *Proceedings of the National Academy of Sciences of the United States of America*.

[111] Darmanis S. (2017). Single-cell RNA-Seq analysis of infiltrating neoplastic cells at the migrating front of human glioblastoma. *Cell Reports*.

[112] Ruiz-Ontañon P., Orgaz J. L., Aldaz B. (2013). Cellular plasticity confers migratory and invasive advantages to a population of glioblastoma-initiating cells that infiltrate peritumoral tissue. *Stem Cells*.

[113] Bao S., Wu Q., McLendon R. E. (2006). Glioma stem cells promote radioresistance by preferential activation of the DNA damage response. *Nature*.

[114] Angelucci C., D’Alessio A., Lama G. (2018). Cancer stem cells from peritumoral tissue of glioblastoma multiforme: the possible missing link between tumor development and progression. *Oncotarget *.

[115] Eidel O., Burth S., Neumann J. (2017). Tumor infiltration in enhancing and non-enhancing parts of glioblastoma: a correlation with histopathology. *PLoS ONE*.

[116] Kelly P. J., Daumas-Duport C., Kispert D. B., Kall B. A., Scheithauer B. W., Illig J. J. (1987). Imaging-based stereotaxic serial biopsies in untreated intracranial glial neoplasms. *Journal of Neurosurgery*.

[117] Martirosyan N. L., Cavalcanti D. D., Eschbacher J. M. (2011). Use of in vivo near-infrared laser confocal endomicroscopy with indocyanine green to detect the boundary of infiltrative tumor. *Journal of Neurosurgery*.

[118] Lemercier P., Maya S. P., Patrie J. T., Flors L., Leiva-Salinas C. (2014). Gradient of apparent diffusion coefficient values in peritumoral edema helps in differentiation of glioblastoma from solitary metastatic lesions. *American Journal of Roentgenology*.

[119] Blystad I., Warntjes J. B., Smedby Ö. (2017). Quantitative MRI for analysis of peritumoral edema in malignant gliomas. *PLoS ONE*.

[120] Chang P. D., Chow D. S., Yang P. H., Filippi C. G., Lignelli A. (2017). Predicting glioblastoma recurrence by early changes in the apparent diffusion coefficient value and signal intensity on FLAIR images. *American Journal of Roentgenology*.

[121] Akbari H., Macyszyn L., Da X. (2016). Imaging surrogates of infiltration obtained via multiparametric imaging pattern analysis predict subsequent location of recurrence of glioblastoma. *Neurosurgery*.

[122] Kallenberg K., Goldmann T., Menke J. (2013). Glioma infiltration of the corpus callosum: early signs detected by DTI. *Journal of Neuro-Oncology*.

[123] Konukoglu E., Clatz O., Bondiau P., Delingette H., Ayache N. (2010). Extrapolating glioma invasion margin in brain magnetic resonance images: Suggesting new irradiation margins. *Medical Image Analysis*.

[124] Nagashima G., Suzuki R., Hokaku H. (1999). Graphic analysis of microscopic tumor cell infiltration, proliferative potential, and vascular endothelial growth factor expression in an autopsy brain with glioblastoma. *Surgical Neurology*.

[125] Pallud J., Varlet P., Devaux B. (2010). Diffuse low-grade oligodendrogliomas extend beyond MRI-defined abnormalities. *Neurology*.

[126] Price S. J., Young A. M., Scotton W. J. (2016). Multimodal MRI can identify perfusion and metabolic changes in the invasive margin of glioblastomas. *Journal of Magnetic Resonance Imaging*.

[127] Baldock A. L., Yagle K., Born D. E. (2014). Invasion and proliferation kinetics in enhancing gliomas predict IDH1 mutation status. *Neuro-Oncology*.

[128] Pérez-Beteta J., Molina-García D., Ortiz-Alhambra J. A. (2018). Tumor surface regularity at MR imaging predicts survival and response to surgery in patients with glioblastoma. *Radiology*.

[129] Jackson P. R., Juliano J., Hawkins-Daarud A., Rockne R. C., Swanson K. R. (2015). Patient-specific mathematical neuro-oncology: using a simple proliferation and invasion tumor model to inform clinical practice. *Bulletin of Mathematical Biology*.

[130] Ellingson B. M., Cloughesy T. F., Lai A., Nghiemphu P. L., Pope W. B. (2011). Cell invasion, motility, and proliferation level estimate (CIMPLE) maps derived from serial diffusion MR images in recurrent glioblastoma treated with bevacizumab. *Journal of Neuro-Oncology*.

[131] Bauer A., Langen K. J., Bidmon H. (2005). 18F-CPFPX PET identifies changes in cerebral A1 adenosine receptor density caused by glioma invasion. *The Journal of Nuclear Medicine*.

[132] Zhao F., Li M., Wang Z. (2015). 18F-Fluorothymidine PET-CT for Resected Malignant Gliomas before Radiotherapy: Tumor Extent according to Proliferative Activity Compared with MRI. *PLoS ONE*.

[133] Harat M., Malkowski B., Wiatrowska I., Makarewicz R., Roszkowski K. (2017). Relationship between glioblastoma dose volume parameters measured by dual time point Fluoroethylthyrosine-PET and clinical outcomes. *Frontiers in Neurology*.

[134] Christensen M., Kamson D. O., Snyder M. (2014). Tryptophan PET-defined gross tumor volume offers better coverage of initial progression than standard MRI-based planning in glioblastoma patients. *Journal of Radiation Oncology*.

[135] Lee I. H., Piert M., Gomez-Hassan D. (2009). Association of 11C-methionine PET uptake with site of failure after concurrent temozolomide and radiation for primary glioblastoma multiforme. *International Journal of Radiation Oncology, Biology, Physics*.

[136] Smith S. J., Diksin M., Chhaya S., Sairam S., Estevez-Cebrero M. A., Rahman R. (2017). The invasive region of glioblastoma defined by 5ALA guided surgery has an altered cancer stem cell marker profile compared to central tumour. *International Journal of Molecular Sciences*.

[137] Stummer W., Pichlmeier U., Meinel T., Wiestler O. D., Zanella F., Reulen H.-J. (2006). Fluorescence-guided surgery with 5-aminolevulinic acid for resection of malignant glioma: a randomised controlled multicentre phase III trial. *The Lancet Oncology*.

[138] Roberts D. W., Valdés P. A., Harris B. T. (2011). Coregistered fluorescence-enhanced tumor resection of malignant glioma: relationships between *δ*-aminolevulinic acid-induced protoporphyrin IX fluorescence, magnetic resonance imaging enhancement, and neuropathological parameters: clinical article. *Journal of Neurosurgery*.

[139] Colditz M. J., Jeffree R. L. (2012). Aminolevulinic acid (ALA)–protoporphyrin IX fluorescence guided tumour resection. Part 1: Clinical, radiological and pathological studies. *Journal of Clinical Neuroscience*.

[140] Hauser S. B., Kockro R. A., Actor B., Sarnthein J., Bernays R. (2016). Combining 5-aminolevulinic acid fluorescence and intraoperative magnetic resonance imaging in glioblastoma surgery. *Neurosurgery*.

[141] Quick-Weller J., Lescher S., Forster M., Konczalla J., Seifert V., Senft C. (2016). Combination of 5-ALA and iMRI in re-resection of recurrent glioblastoma. *British Journal of Neurosurgery*.

[142] Kiesel B., Mischkulnig M., Woehrer A. (2018). Systematic histopathological analysis of different 5-aminolevulinic acid–induced fluorescence levels in newly diagnosed glioblastomas. *Journal of Neurosurgery*.

[143] Jenkinson M. D., Barone D. G., Bryant A. (2018). Intraoperative imaging technology to maximise extent of resection for glioma. *Cochrane Database of Systematic Reviews*.

[144] Stockhammer F., Misch M., Horn P., Koch A., Fonyuy N., Plotkin M. (2009). Association of F18-fluoro-ethyl-tyrosin uptake and 5-aminolevulinic acid-induced fluorescence in gliomas. *Acta Neurochirurgica*.

[145] Roessler K., Becherer A., Donat M., Cejna M., Zachenhofer I. (2012). Intraoperative tissue fluorescence using 5-aminolevolinic acid (5-ALA) is more sensitive than contrast MRI or amino acid positron emission tomography ((18)F-FET PET) in glioblastoma surgery. *Neurological Research*.

[146] Floeth F. W., Sabel M., Ewelt C. (2011). Comparison of 18F-FET PET and 5-ALA fluorescence in cerebral gliomas. *European Journal of Nuclear Medicine and Molecular Imaging*.

[147] Neira J. A., Ung T. H., Sims J. S. (2017). Aggressive resection at the infiltrative margins of glioblastoma facilitated by intraoperative fluorescein guidance. *Journal of Neurosurgery*.

[148] Yano H., Nakayama N., Ohe N., Miwa K., Shinoda J., Iwama T. (2017). Pathological analysis of the surgical margins of resected glioblastomas excised using photodynamic visualization with both 5-aminolevulinic acid and fluorescein sodium. *Journal of Neuro-Oncology*.

[149] Moiyadi A. V., Shetty P. M., Mahajan A., Udare A., Sridhar E. (2013). Usefulness of three-dimensional navigable intraoperative ultrasound in resection of brain tumors with a special emphasis on malignant gliomas. *Acta Neurochirurgica*.

[150] Solheim O., Selbekk T., Jakola A. S., Unsgård G. (2010). Ultrasound-guided operations in unselected high-grade gliomas—overall results, impact of image quality and patient selection. *Acta Neurochirurgica*.

[151] Del Bene M. (2018). Advanced ultrasound imaging in glioma surgery: beyond gray-scale B-mode. *Frontiers in Oncology*.

[152] Prada F., Mattei L., Bene M. D. (2014). Intraoperative cerebral glioma characterization with contrast enhanced ultrasound. *BioMed Research International*.

[153] Lindner D., Trantakis C., Renner C. (2006). Application of intraoperative 3D ultrasound during navigated tumor resection. *Minimally Invasive Neurosurgery*.

[154] Prada F., Vitale V., Del Bene M. (2017). Contrast-enhanced MR imaging versus contrast-enhanced US: a comparison in glioblastoma surgery by using intraoperative fusion imaging. *Radiology*.

[155] Sanai N., Eschbacher J., Hattendorf G. (2011). Intraoperative confocal microscopy for brain tumors: a feasibility analysis in humans. *Operative Neurosurgery*.

[156] Schlosser H., Suess O., Vajkoczy P., Landeghem F. K., Zeitz M., Bojarski C. (2010). Confocal Neurolasermicroscopy in Human Brain – Perspectives for Neurosurgery on a Cellular Level (including additional Comments to this article). *Central European Neurosurgery*.

[157] Sankar T., Delaney P. M., Ryan R. W. (2010). Miniaturized handheld confocal microscopy for neurosurgery. *Neurosurgery*.

[158] Yashin K. S., Kiseleva E. B., Moiseev A. A. (2019). Quantitative nontumorous and tumorous human brain tissue assessment using microstructural co- and cross-polarized optical coherence tomography. *Scientific Reports*.

[159] Kut C., Chaichana K. L., Xi J. (2015). Detection of human brain cancer infiltration ex vivo and in vivo using quantitative optical coherence tomography. *Science Translational Medicine*.

[160] Yuan W., Kut C., Liang W., Li X. (2017). Robust and fast characterization of OCT-based optical attenuation using a novel frequency-domain algorithm for brain cancer detection. *Scientific Reports*.

[161] Böhringer H. J., Lankenau E., Stellmacher F., Reusche E., Hüttmann G., Giese A. (2009). Imaging of human brain tumor tissue by near-infrared laser coherence tomography. *Acta Neurochirurgica*.

[162] Lefranc F., Le Rhun E., Kiss R., Weller M. (2018). Glioblastoma quo vadis: Will migration and invasiveness reemerge as therapeutic targets?. *Cancer Treatment Reviews*.

[163] Mandonnet E., Capelle L., Duffau H. (2006). Extension of paralimbic low grade gliomas: toward an anatomical classification based on white matter invasion patterns. *Journal of Neuro-Oncology*.

[164] Chaichana K. L., Jusue-Torres I., Navarro-Ramirez R. (2014). Establishing percent resection and residual volume thresholds affecting survival and recurrence for patients with newly diagnosed intracranial glioblastoma. *Neuro-Oncology*.

[165] Xu D. S., Awad A., Mehalechko C. (2018). An extent of resection threshold for seizure freedom in patients with low-grade gliomas. *Journal of Neurosurgery*.

[166] Li Y. M., Suki D., Hess K., Sawaya R. (2016). The influence of maximum safe resection of glioblastoma on survival in 1229 patients: Can we do better than gross-total resection?. *Journal of Neurosurgery*.

[167] Yan J., van der Hoorn A., Larkin T. J., Boonzaier N. R., Matys T., Price S. J. (2017). Extent of resection of peritumoral diffusion tensor imaging–detected abnormality as a predictor of survival in adult glioblastoma patients. *Journal of Neurosurgery*.

[168] Pessina F., Navarria P., Cozzi L. (2017). Maximize surgical resection beyond contrast-enhancing boundaries in newly diagnosed glioblastoma multiforme: is it useful and safe? A single institution retrospective experience. *Journal of Neuro-Oncology*.

[169] Elson A., Paulson E., Bovi J., Siker M., Schultz C., Laviolette P. S. (2015). Evaluation of pre-radiotherapy apparent diffusion coefficient (ADC): patterns of recurrence and survival outcomes analysis in patients treated for glioblastoma multiforme. *Journal of Neuro-Oncology*.

[170] Grossman R., Shimony N., Shir D. (2017). Dynamics of FLAIR volume changes in glioblastoma and prediction of survival. *Annals of Surgical Oncology*.

[171] Mampre D., Ehresman J., Pinilla-Monsalve G. (2016). Extending the resection beyond the contrast-enhancement for glioblastoma: feasibility, efficacy, and outcomes. *British Journal of Neurosurgery*.

[172] Pei J., Park I., Ryu H. (2015). Sublethal dose of irradiation enhances invasion of malignant glioma cells through p53-MMP 2 pathway in U87MG mouse brain tumor model. *Journal of Radiation Oncology*.

[173] Zaboronok A., Isobe T., Yamamoto T. (2014). Proton beam irradiation stimulates migration and invasion of human U87 malignant glioma cells. *Journal of Radiation Research*.

[174] Minniti G., Amelio D., Amichetti M. (2010). Patterns of failure and comparison of different target volume delineations in patients with glioblastoma treated with conformal radiotherapy plus concomitant and adjuvant temozolomide. *Radiotherapy & Oncology*.

[175] Chang E. L., Akyurek S., Avalos T. (2007). Evaluation of peritumoral edema in the delineation of radiotherapy clinical target volumes for glioblastoma. *International Journal of Radiation Oncology, Biology, Physics*.

[176] Cabrera A. R., Kirkpatrick J. P., Fiveash J. B. (2016). Radiation therapy for glioblastoma: executive summary of an american society for radiation oncology evidence-based clinical practice guideline. *Practical Radiation Oncology*.

[177] Wernicke A., Smith A. W., Taube S., Mehta M. P. (2016). Glioblastoma: Radiation treatment margins, how small is large enough?. *Practical Radiation Oncology*.

[178] Berberat J., McNamara J., Remonda L., Bodis S., Rogers S. (2014). Diffusion tensor imaging for target volume definition in glioblastoma multiforme. *Strahlentherapie und Onkologie*.

[179] Jena R., Price S. J., Baker C. (2005). Diffusion tensor imaging: possible implications for radiotherapy treatment planning of patients with high-grade glioma. *Clinical Oncology Journal (The Royal College of Radiologists)*.

[180] Jensen M. B., Guldberg T. L., Harbøll A., Lukacova S., Kallehauge J. F. (2017). Diffusion tensor magnetic resonance imaging driven growth modeling for radiotherapy target definition in glioblastoma. *Acta Oncologica*.

[181] Harat M., Małkowski B., Makarewicz R. (2016). Pre-irradiation tumour volumes defined by MRI and dual time-point FET-PET for the prediction of glioblastoma multiforme recurrence: A prospective study. *Radiotherapy & Oncology*.

[182] Oehlke O., Mix M., Graf E. (2016). Amino-acid PET versus MRI guided re-irradiation in patients with recurrent glioblastoma multiforme (GLIAA) – protocol of a randomized phase II trial (NOA 10/ARO 2013-1). *BMC Cancer*.

[183] Wick W., Wick A., Schulz J. B., Dichgans J., Rodemann H. P., Weller M. (2002). Prevention of irradiation-induced glioma cell invasion by temozolomide involves caspase 3 activity and cleavage of focal adhesion kinase. *Cancer Research*.

[184] Hombach-Klonisch S., Mehrpour M., Shojaei S. (2018). Glioblastoma and chemoresistance to alkylating agents: Involvement of apoptosis, autophagy, and unfolded protein response. *Pharmacology & Therapeutics*.

[185] Sarkaria J. N., Hu L. S., Parney I. F. (2018). Is the blood–brain barrier really disrupted in all glioblastomas? A critical assessment of existing clinical data. *Neuro-Oncology*.

[186] Goldwirt L., Beccaria K., Carpentier A. (2015). Preclinical impact of bevacizumab on brain and tumor distribution of irinotecan and temozolomide. *Journal of Neuro-Oncology*.

[187] Safari M., Khoshnevisan A. (2015). Cancer stem cells and chemoresistance in glioblastoma multiform: A review article. *Journal of Stem Cells*.

[188] Keunen O., Johansson M., Oudin A. (2011). Anti-VEGF treatment reduces blood supply and increases tumor cell invasion in glioblastoma. *Proceedings of the National Academy of Sciences of the United States of America*.

[189] Rahman M., Azari H., Deleyrolle L., Millette S., Zeng H., Reynolds B. A. (2013). Controlling tumor invasion: bevacizumab and BMP4 for glioblastoma. *Future Oncology*.

[190] Xie Q., Mittal S., Berens M. E. (2014). Targeting adaptive glioblastoma: An overview of proliferation and invasion. *Neuro-Oncology*.

